# Tympanic Pre-Operative Electrically Evoked Auditory Late Response (TympEALR) as an Alternative to Trans-Tympanic Tests Using Anesthesia in Cochlear Implant Candidacy

**DOI:** 10.3390/jcm13247573

**Published:** 2024-12-12

**Authors:** Daniel Polterauer, Maike Neuling, Florian Simon

**Affiliations:** Section Cochlear Implantation, Department of Otorhinolaryngology, University Hospital of Munich (LMU), 81377 Munich, Germany; maike.neuling@med.uni-muenchen.de (M.N.); florian.simon@med.uni-muenchen.de (F.S.)

**Keywords:** cochlear implantation, evoked potential, hearing, EABR, EAMLR, EALR, auditory pathway

## Abstract

**Background/Objectives**: Before a cochlear implant is considered, patients undergo various audiological tests to assess their suitability. One key test measures the auditory brainstem response (ABR) to acoustic stimuli. However, in some cases, even with maximum sound stimulation, no response is detected. **Methods**: The promontory test involves electrical stimulation near the auditory nerve, allowing patients to associate the sensation. Ideally, the electrode is placed in the middle ear after opening the eardrum. This method, along with trans-tympanic electrically evoked ABR in local anesthesia (LA-TT-EABR) and the cortical equivalent (LA-TT-EALR), helps assess the auditory nerve’s existence and excitability. The TympEALR test, utilizing a “tympanic LA-TT-EALR”, provides an alternative measurement. Previous research has shown the possibility of deriving brainstem and cortical potentials through trans-tympanic electrical stimulation, allowing for objective assessment of the auditory nerve’s integrity and potentially objectifying patient sensations. **Results:** Sixteen patients have been tested using TympEALR. In seven of these, we found a positive response. The morphology was similar to other electrically evoked cortical auditory responses (EALR), e.g., using cochlear implants or trans-tympanic stimulation electrodes. We observed a higher influence of electrical artifacts than in other EALRs. **Conclusions**: TympEALR showed positive results in nearly half of the study participants, potentially avoiding invasive procedures. TympEALR can be a valuable alternative to trans-tympanic methods. More research is needed to determine if a negative result suggests against cochlear implantation.

## 1. Introduction

Over the past decades, starting with the first trials in 1951 in France by Charles Eyries and Andre Djourno and the single-channel implants of Dr. William House in 1965 in the United States [1,2], the indication of cochlear implantation—being the most successful active prosthesis stimulating a cranial nerve—has been changed over and over again. In the early days, only completely deaf patients were considered for a cochlear implant (=CI) on one ear [3]. Today, more and more patients with better hearing are considered for a CI due to its great success [4]. The hearing loss severity after CI implantation moved from complete deafness in both ears to severe hearing loss (based on a national consensus) in one ear [5]. Nowadays, patient groups that have a high residual hearing in the low-frequency spectrum are possible CI candidates. This is enabled by soft intracochlear electrode arrays, different types of these electrode arrays (especially straight vs. pre-curved and forms for malformed cochleae), different lengths of these electrode arrays to cover the wide range of cochlear sizes across patients’ individual anatomy, new surgical and robotic techniques and also live monitoring of the residual hearing of the lower frequencies during the insertion of the electrode array in the cochlear duct [6,7,8,9,10,11,12,13,14,15,16,17,18,19]. This special group with residual hearing in the lower frequencies can receive so-called electro-acoustic-stimulation processors stimulating the lower frequencies with an integrated hearing aid and the mid to high frequencies electrically using the electrode array [20]. Also, patients with very little word understanding can be considered for CI though their hearing threshold alone would indicate a hearing aid instead.

However, there is a trend of implanting patients with a CI who have much better hearing than CI users had some decades ago. In contrast, patients with insecure CI candidacy are implanted with CI more often [21,22]. The success of hearing and speech understanding after cochlear implantation is questionable, additional measurements can be performed to check the eligibility of these candidates and avoid a frustrating future for patients and clinicians [23,24].

During the pre-investigations for cochlear implantation, numerous audiological tests are conducted to determine the suitability of the respective patient for a CI as well as careful evaluation of the anesthesiology according to the newest guidelines [25]. Among these tests, the auditory brainstem response to acoustic stimuli is used, mainly through auditory brainstem response (=ABR) recording. In some patients, even with maximum acoustic stimulation, no response can be detected. An alternative is the promontory test, where the patient is electrically stimulated near the auditory nerve, which is associated with a sensation. Ideally, the electrode is placed in the middle ear for electrical stimulation after the tympanic membrane is opened. In this trans-tympanic setup, electrically evoked ABR (=EABR), electrically evoked auditory mid-late response (=EAMLR), and electrically evoked auditory late response (=EALR) can be recorded when using local anesthesia similar to results from intra-cochlear test electrodes like the auditory nerve test electrode “ANTS” or the gold standard cochlear implant stimulation [26,27,28,29,30,31,32]. Using general anesthesia offers EABR [33] and EAMLR [34,35,36] while EALR can only be recorded in awake patients. Trans-tympanic EABR, EALMR, and EALR (TT-EABR resp. TT-EAMLR resp. TT-EALR) are useful tools to examine the existence of the auditory nerve and thus, the excitability of the auditory pathway [33,34,35,37,38,39,40,41,42,43,44,45]. Compared to a trans-tympanic promontory test, only a small additional effort is required when performing a tympanic promontory test. However, both options allow the activation of the auditory cortex as shown in fMRI research [46]. Previous results have shown that deriving brainstem potentials and cortical potentials is possible with trans-tympanic electrical stimulation. It allows for the objective integrity assessment of the auditory nerve. Insecure sensations of the patients can potentially be objectified in this way. Now, an alternative measurement is to be carried out with the help of a tympanic PromCERA, the so-called “TympEALR” also known as the “PromCERA light” [47]. Choosing EALR, the auditory pathway can be analyzed up to the cortex objectively [48] with fewer interfering stimulation artifacts, myogenic artifacts, or facial nerve responses compared to EABR recording [49].

This study aimed to initially evaluate the TympEALR retrospectively in a small collective to find out whether it is possible to record responses similar to those known from other EALR measurements. In addition, we were interested in the subjective sensations across our patient collective. Finally, the study hypothesized that TympEALR may contribute to pre-operative diagnostics where acoustic AEP failed without the need for anesthetics and the paracentesis in trans-tympanic electrically evoked auditory potentials like LA-TT-EABR.

## 2. Materials and Methods

### 2.1. Study Design and Data Collection

This observational retrospective mono-centric study included all patients from 2018 to 2022 that were tested using the TympEALR. Clinical data were analyzed, which included the patient’s age, sex, date of the TympEALR, position of the stimulation electrodes, subjective sensation(s) during TympEALR testing (including its location), subjective levels of sensation (UCL, MCL, SL), stimulation resistance before and after TympEALR testing, result of the TympEALR testing (positive, insecure, or negative), etiology of hearing loss, duration of hearing loss, duration of deafness, TympEALR response amplitude (N1-N2/P3), and latencies (N1, P2, P3, N2). Patients who did not meet the inclusion criteria had a perforated ear drum, inflammation of the ear canal, or intolerance to the materials of the ear canal electrode or the surface electrodes for stimulation or recording.

Data export was performed anonymized using Innoforce ENTstatistics (Ruggell, Liechtenstein) overall patient export tool. This concept was approved by our university ethics commission (project number 24-0923).

We performed TympEALR recording parallel to standard tympanic promontory nerve test in 16 cochlear implant candidates at our university hospital ORL department. These patients were tested by TympEALR when we found no or questionable response in acoustic AEP and had no or questionable hearing according to the tone audiogram as well as no or questionable speech understanding in the Freiburg monosyllable and Freiburg number test. The TympEALR replaced them in our clinic traditionally performed tympanic subjective “promontory test” during hearing tests before cochlear implant surgery.

### 2.2. Technical Setup for the TympEALR

**Figure 1:**
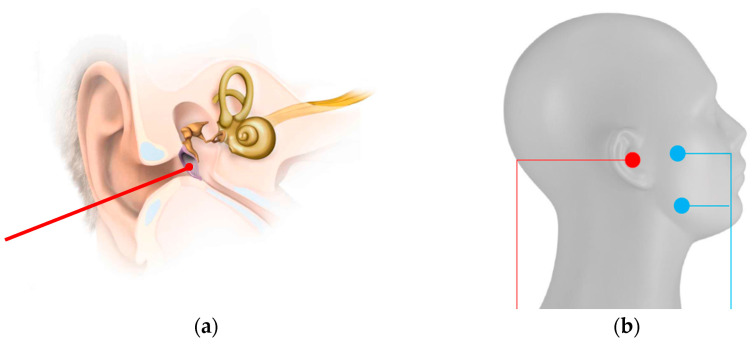
Stimulation setup for TympEALR: (**a**) Position of the active electrode (red) in the outer ear canal on the tympanic membrane. (**b**) Position of the counter electrodes (blue) on the mandibular and zygomatic arch relative to the active electrode (red). (Image sources: MED-EL, Innsbruck; modified).

For TympEALR, we used established systems for stimulation and measurement of evoked potentials. For the stimulation, we used the electro-stimulator “inomed Neurostimulator ISIS”. We recorded the response signals by the EP system “Nihon Kohden Neuropack S1 MEB-9400”. A BNC-Trigger cable (TTL 5V signal) was connected between the electro-stimulator and the EP system to enable the synchronization of stimulation impulses and timing of response recording. The stimulation counter electrodes (“ambu neuroline 720-00s”) were positioned on the mandibular and zygomatic arch short-circuited with each other as known from trans-tympanic EABR, EAMLR, and EALR in local anesthesia [37,38,43]. One out of twelve medical doctors put the active stimulation electrode (“Sanibel TM electrode” or ”inomed disposable Tympanon electrode 530 453”) on the tympanic membrane. To ease the insertion of the tympanic electrode, it was surrounded by electrode gel manually (Parker signa gel). The stimulation setup for TympEALR is shown in Figure 1.

Initially, as a pre-test, an electrostimulation without a recording of evoked potentials was performed as known from a conventional tympanic promontory test. As standard stimulation parameters for the subjective pre-test, we selected a 50 Hz stimulation frequency using a biphasic pulse of 10.5 ms pulse width per phase (i.e., 21 ms full pulse width). Before the stimulation, a stimulation impedance test integrated into the electro-stimulator was performed to ensure proper contact with the tissue. By this, we collected data about the subjective sensation(s) given by the electrical stimulation (no sensation, hearing, pain, uncomfortable feeling, other, or unknown sensation) and the area of sensation (ear, face, throat/neck, or other). In addition, individual stimulation levels for the threshold (SL), the maximum comfortable level (MCL), and the uncomfortable level (UCL) were acquired in the health records.

For TympEALR, we expected a similar EALR as known from trans-tympanic or cochlear implant stimulation [43,48,50]. Therefore, the stimulation frequency was reduced to 0.9 Hz to enable the recording of the desired time window after stimulation in the EEG. To be able to record responses we chose alternating polarity. Alternating polarity is known to reduce artifacts, especially the stimulation artifact in EPs when using electrical stimulators [51]. Because of software restrictions, the electro-stimulator “inomed Neurostimulator ISIS” was not able to use alternating polarity. Therefore, we recorded alternating polarity by stimulation with a positive initial phase first and with a negative initial phase second. The mean of these two curves gave us one manually created alternating polarity waveform. To record during tympanic electrical stimulation, we used the clinical recording setting for EALR in our experimental attempt (see Table 1).

To differentiate between clear response (=positive TympEALR), unclear response, and absent response (=negative TympEALR) the mean of 2 to 3 manually averaged waveforms was visually analyzed for ipsilateral and contralateral recording. As there is not enough data about response amplitude in preoperative EALR, we chose postoperative EALR as a reference. Postoperative EALR in adult cochlear implant patients as well as basal ALR in normal hearing adults show N1-P2 response amplitudes at 3 µV or more like it is chosen in ALR according to Guy Lightfoot and there is no evidence that EALR differs much according to Hughes [52,53]. Therefore, a lower limit of 3 µV was set for response amplitudes in TympEALR. The response latencies in preoperative EALR are N1, P2, P3 (not always clearly separate from P2), and N2 which is located at 100 ms for N1 and 200-300 ms for N2 with P2 and sometimes P3 around 150–200 ms [43]. We chose to use these values as a reference for the detection of TympEALR with an acceptance window of ±50 ms for each latency marker. In summary, a response of EALR morphology and 3 µV response amplitude is called a positive TympEALR. If a response is of EALR morphology but the response amplitude is lower than 3 µV it is called an insecure TympEALR. If the response is not of EALR morphology it is called a negative TympEALR.

If ipsilateral and contralateral mean waveforms were matching in morphology similar to other EALR and the amplitude of N1-P2/P3 was ≥3 µV we defined the result as a positive TympEALR. If the morphology was matching but the amplitude was <3 µV we defined the result as an insecure TympEALR. All other cases were defined as negative TympEALR.

### 2.3. Statistical Analysis

The data analysis for this paper was generated using the Real Statistics Resource Pack software (Release 7.9.1). Copyright (2013–2024) Charles Zaiontz. www.real-statistics.com (accessed on 29 November 2021). Numerical values were tested for normal distribution using the Shapiro–Wilk test. The Pearson Correlation test was chosen for normally distributed values. For not-normally distributed, we chose the Spearman correlation test. Probability values of *p* < 0.05 were considered significant.

## 3. Results

In one of the 16 patients included in this study, we performed TympEALR on both sides during one session. The other patients were tested solely unilaterally. Seven right ears and ten left ears were tested. Seven patients were male and nine patients were female. When we performed TympEALR the mean age was 46.94 ± 17.48 years with a range from 16 years to 77 years of age.

In eight patients, the etiology of their deafness was unknown. The bilaterally tested patient was deafened by toxic antibiotic treatment. Two patients were deafened by a vestibular schwannoma and one by vestibular schwannoma resection. Two patients were deafened by meningitis. One patient was deafened after an infection with herpes zoster. One patient was deafened by a traumatic brain trauma.

The mean duration of hearing loss was 13.54 ± 17.66 years with a minimum of 0.25 years and a maximum of 63 years. The mean duration of deafness was 9.84 ± 12.66 years with a minimum of 0.25 years and a maximum of 47 years.

The patients’ histories of hearing including their age at TympEALR testing, the tested ear by TympEALR, the etiology of hearing loss/deafness, and their duration of hearing loss as well as of deafness are in Table 2.

All patients reported some kind of subjective sensation during the pre-test or the recording of TympEALR. An overview of the patients’ sensations is shown in Table 3. Mostly an unknown sensation in the ear was reported (N = 12). If a patient reported “other sensation”, we additionally noted his or her individual description. Among these seven cases, four times some kind of pressure feeling was reported, once a vibration and once a temperature change. All these “other sensations” were located in the ear. The one patient tested bilaterally reported an unknown sensation in both ears.

**Figure 2:**
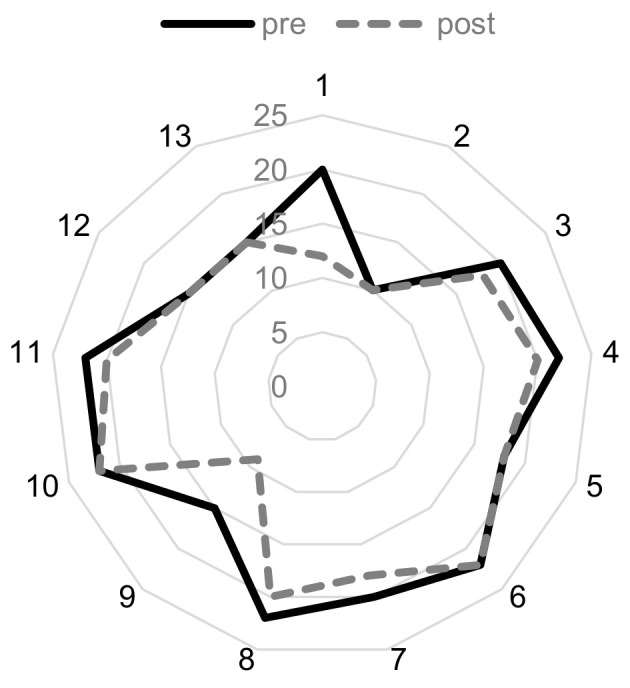
Comparison of the individual stimulation impedance in kΩ before the first stimulation at pre-testing (=pre; solid line) and after the last TympEALR recording (=post; dashed line).

We analyzed the data about the subjective stimulation levels and stimulation impedances. The mean UCL was 187.69 ± 78.66 µA with a minimum of 60 µA and a maximum of 300 µA (N = 14). The mean MCL was 170.00 ± 68.87 µA with a minimum of 50 µA and 280 µA (N = 15). The SL was 107.33 ± 59.27 with a minimum of 20 µA and a maximum of 200 µA (N = 16). The mean stimulation impedance before the first stimulation at pre-testing was 19.00 ± 3.67 kΩ with a minimum of 10 kΩ and a maximum of 22 kΩ and 16.00 ± 4.02 kΩ with a minimum of 9 kΩ and a maximum of 20 kΩ after the last TympEALR recording (N = 13, see Figure 2).

### 3.1. Positive, Insecure, or Negative TympEALR

In seven cases we found a positive response in TympEALR recording. In nine ears including both sides of the bilaterally tested patient, we found an insecure response. In the one remaining case, we found no clear response maybe caused by unusually high EEG variation and a very prominent stimulus artifact in the EP recording.

For the positive TympEALR cases, we found a response amplitude N1-P2/P3 of 4.64 ± 1.94 µA with a minimum of 3 µV and a maximum of 8.9 µV. The latency response for N1 was 90.04 ± 23.00 ms with a minimum of 55 ms and a maximum of 120 ms. The latency response for P2 was 146.52 ± 42.11 ms with a minimum of 108 ms and a maximum of 229.5 ms. In two cases, we found P3 with a latency of 185.37 ms in one case and 180.5 in the other case. The latency response for N2 was 351.5 ± 59.15 with a minimum of 166 ms and a maximum of 351.5 ms.

For insecure TympEALR cases, we found a response amplitude N1-P2/P3 of 1.79 ± 0.84 µA with a minimum of 0.41 µV and a maximum of 2.9 µV. The latency response for N1 was 89.17 ± 23.70 ms with a minimum of 59.72 ms and a maximum of 131.5 ms. The latency response for P2 was 137.43 ± 30.26 ms with a minimum of 86.84 ms and a maximum of 200.5 ms. In five cases, we found P3 with a latency of 182.36 ± 28.45 ms with a minimum of 156.86 ms and a maximum of 236 ms. The latency response for N2 was 226.18 ± 52.13 ms with a minimum of 172.55 ms and a maximum of 349.5 ms.

An overview of all TympEALR showing N1-P2 resp. N1-P3 response amplitude and the latencies of N1, P2, P3, and N2 can be found in Table 4 for each individual patient.

**Figure 3:**
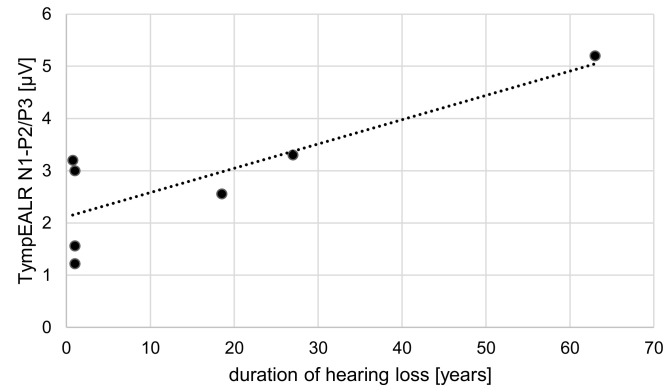
Scatter plot showing the correlation between the duration of hearing loss in years and the amplitude of N1-P2/P3 from the TympEALR in µV.

A correlation test was performed for the duration of hearing loss vs. N1-P2/P3 response amplitude and for the duration of deafness vs. N1-P2/P3 response amplitude. The Pearson correlation coefficient assesses the linear relationship between the duration of hearing loss and N1-P2/P3 amplitude. There was a positive correlation between the two variables, r(5) = 0.83, *p* = 0.02 (see Figure 3). The Pearson correlation coefficient was computed to assess the linear relationship between the duration of deafness and N1-P2/P3 amplitude. There is no significant correlation between the two variables, r(4) = 0.45, *p* = 0.38.

**Figure 4:**
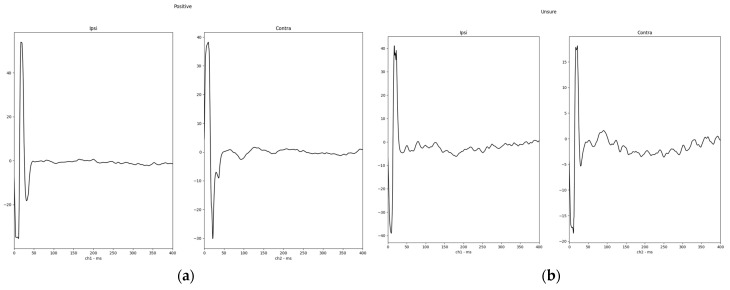
Total mean of response waveforms for study patients with a (**a**) positive TympEALR (typical EALR latencies and a minimum N1-P2/P3 response amplitude of 3 µV, *n* = 7); (**b**) insecure TympEALR (typical EALR latencies and a N1-P2/P3 response amplitude of less than 3 µV, *n* = 9).

**Figure 5:**
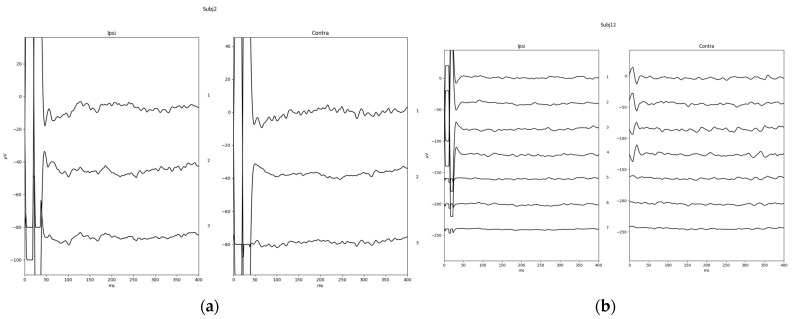
Single cases of a TympEALR (**a**) a positive response including all measurements (positive and negative initial stimulation phase (1st and 2nd waveforms from top) and manually averaged response (3rd waveform from top) (subject 02); (**b**) the only negative TympEALR in our study group (subject 12). For subject 02 and subject 12, recording channel 1 (left curves) was the contralateral side of testing = right mastoid recording electrode, and recording channel 2 (right curves) was the ipsilateral dies of testing = left mastoid recording electrode.

Finally, to show a typical response waveform, a total mean was plotted from a direct export of the response waveform selected as described before. The total mean of the response waveforms grouped in positive and insecure are shown in Figure 4. One example of a positive response (subject 02) and the only negative response in the investigated study group (subject 12) are shown in Figure 5.

### 3.2. Clinical Procedure After TympEALR Testing

Eight of these seventeen ears (four positive, three insecure, and one negative TympEALR) including both sides of the bilaterally tested patient received a cochlear implant. As we did not know the reliability of TympEALR results the decision pro or contra CI was only influenced by TympEALR. Other than TympEALR, other arguments like radiology results in CT and MRI or the individual willingness of the patient contributed to the final decision of whether a patient was implanted or not. All eight patients were having hearing sensations with their cochlear implant systems. Seven also had a speech understanding. Six months after the initial cochlear implant activation, the Freiburg monosyllable word recognition score at 65dB SPL in quiet was 41.25 ± 29.02% with a minimum of 0% and a maximum of 70%. There were two patients with no word recognition six months after cochlear implant activation. The one patient (positive TympEALR) with a 0%-word recognition score six months after initial CI activation (later data are missing) could understand items in the Freiburg multi-syllable number test. Another patient (positive TympEALR) with a 0%-word recognition score one month after initial CI activation (later speech understanding was not documented in our database) also could not understand items in the Freiburg multi-syllable number test but was hearing tones using the CI.

**Figure 6:**
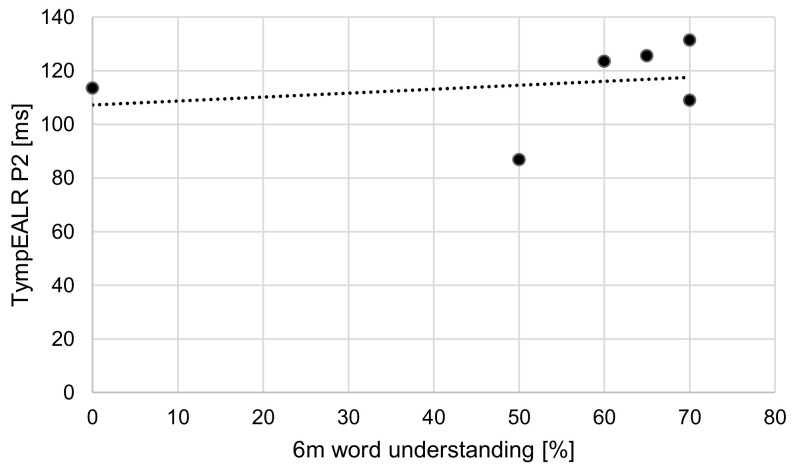
Scatter plot showing the correlation between the word understanding six months after first fitting of the cochlear implant system in percent and the latency of P2 from the TympEALR in ms.

The Pearson correlation coefficient was computed to assess the linear relationship between word understanding six months after the first fitting of the cochlear implant system and P2 latency. There is no significant correlation between the two variables, r(4) = −0.25, *p* = 0.64. The data are shown in Figure 6.

## 4. Discussion

In the past, the subjective promontory stimulation test was used to evaluate the indication of CI in insecure candidates (especially in retro-cochlear etiologies). Due to the low specificity of the subjective promontory test, it is used rarely today. With trans-tympanic EABR an objective promontory test was found as a good alternative to the subjective promontory test. The testing in local anesthesia offers an interesting setup excluding general anesthesia and includes the possibility to record EAMLR and EALR in addition and being able to assess the patient’s subjective sensation during stimulation. Based on the data presented in this study, the TympEALR looks like another step forward as no anesthesia is needed at all.

To the best of our knowledge, this study is the first to utilize TympEALR or similar measurements. Therefore, the small number of patients gives us a limited possibility to compare the reliability of TympEALR with the trans-tympanic equivalent in local anesthesia (LA-TT-EALR) or measurements of brainstem resp. midbrain responses in local or general anesthesia (TT-EABR/EAMLR). A larger dataset is needed to finally prove the concept of TympEALR and compare it, especially to LA-TT-EALR as a pre-test checking for a clear response in TympEALR and other cases going for LA-TT-EALR. In the case of insecure or negative LA-TT-EALR, LA-TT-EABR and LA-TT-EAMLR can be assessed in the same session within a short time.

Other than the mentioned benefits of TympEALR to objective promontory tests using the classical trans-tympanic approach, we need to address also the critical points. First, the stimulation is delivered from the tympanic membrane instead of the area around the round window niche. By this, there is a need for a higher stimulation charge to stimulate the auditory path which enlarges the stimulation artifact in the recorded waveforms and additionally the possibility and strength of co-stimulations (e.g., facial nerve). The enlarged stimulation artifact brought us to solely being able to record TympEALR from the EEG and impeded the recording of TympEABR. In future studies, TympEAMLR could be evaluated. The enlarged co-stimulation in TympEALR is limiting the maximum tolerance level of stimulation charge. This potentially leads to a non-positive TympEALR though the auditory pathway is electrically excitable. In addition, this effect is in line with the several unsecure subjective sensations at TympEALR reported within our study. In the cases with non-positive TympEALR, LA-TT-EALR would be the next logical step.

In the case of TympEALR, the electrical stimulation is delivered from the tympanic membrane to the auditory nerve. In the case of LA-TT-EABR/-EAMLR/-EALR, the stimulation is delivered from the round window niche to the auditory nerve. As LA-TT-EABR stimulating in the round windows niche is known to show waveforms like “very basal” EABR using CI stimulation, we hypothesize that TympEALR stimulating “even more basal” shows waveforms like an “even more basal” CI stimulation. This leads us to the limiting factor known from CI stimulation evoked potentials, i.e., EABR, EAMLR, and EALR: The more apical the stimulation the lower the response latency and the larger the amplitudes of the response waveforms [54]. Luckily, EALRs are known to give relatively large response waveforms also slightly above the threshold which for sure is a big factor why TympEALR works. As no cognitive impairment was known in our tested patients within this study no reduction in response amplitude can be assumed [55].

By looking at stimulation impedances, we found stability or a slight reduction over the time of TympEALR (before vs. after testing). This finding is similar to the results known from trans-tympanic testing and also with experiences from subjective tympanic promontory testing. All over, the stimulation in TympEALR seems to be stable and in regular cases, no continuous checking of stimulation impedance should be needed.

In our study dataset, we checked for correlations of hearing loss vs. N1-P2/P3 response amplitude and duration of deafness vs. N1-P2/P3 response amplitude. For the duration of hearing loss, a positive linear relationship was found. In contrast, for the duration of deafness, no significant correlation was found. First, we have to keep in mind the relatively small dataset of our study and the variability within patients’ demography. Secondly, the correlation of hearing loss relies on several complex etiologies like vestibular schwannoma (resection). Therefore, this significant correlation needs to be re-evaluated in future studies.

The postoperative word recognition tests after cochlear implantation focused on the one patient with no speech understanding (no words and no Freiburg multisyllables). Though later data are missing (only one month after the initial cochlear implant fitting session was available) this patient had the highest latency for P2 (i.e., 229.50 ms) and also the highest latency for N2 (i.e., 351.50 ms) which could indicate that the response may not be auditory. The patient had a sudden hearing loss due to a traffic accident followed by a dens axis fracture and received a SYNCHRONY 2 S-VECTOR (Mi1250) with a STANDARD electrode array (compare estimated preoperative CT-based cochlear duct length of 33.7 mm [19,56]). During surgery, impedance measurements showed that contact between the stimulation electrodes of the CI and the cochlear tissue was in the normal range. No electrically evoked compound action potentials could be recorded nor could electrically evoked stapedius reflexes be recognized. Due to this, we also performed an intra-operative eABR using the test electrode system “ANTS” giving a reproducible wave V and therefore, confirmed the connection of the CI to the patient’s auditory brainstem [27,57]. Other than the missing word and number understanding, the patient achieved hearing and the discrimination of tones. On the non-implanted ear, the patient had a CPT-AMA of 13.1%. Therefore, single-sided deafness may also be part of the problem as chances for achieving speech understanding can vary a lot when comparing different hearing histories/etiologies [58]. Most likely, we suppose that the trauma causing the single-sided deafness also caused an auditory neuropathy. In those cases, instead of traumatic damage of the synaptic connection at the modiolus, poor performance with cochlear implants is reported [59].

Most of all ORL departments should be easily able to establish TympEALR in a clinical routine for individual cases. The stimulation system or a similar one with trigger output should be available as subjective promontory testing is an established method and the same stimulator can be used. The recording EP device used in this study may be replaced by another EP device having good shielding against electrical stimulation artifacts. The setup of TympEALR is almost as easy as subjective promontory testing and should therefore be possible in any ORL.

## 5. Limitations

Some factors are limiting this study’s results. At first, this is, as far as we know, the first study about a tympanic EALR. Therefore, we selected simulation values and patients for TympEALR as if patients were selected for the subjective tympanic promontory testing. For recording, we used established values known for other EALR measurements using the trans-tympanic approach or for stimulation by a cochlear implant. This is not an optimized setup for TympEALR and may be improved in future testing. A different setup for stimulation like an electric burst instead of a single biphasic pulse or different band-pass filtering for recording could be possible. In addition, future studies may test different electrode locations for stimulation and also recording to optimize the TympEALR.

Due to the retrospective design of this study, we do not have all the data for each subject (e.g., speech tests post-operatively) and only a small number of patients were tested. Furthermore, we must rely on the accuracy of recordkeeping. This small number of patients results in a limited statistical power. We also do not have the available data for TympEALR and LA-TT-EALR to compare the tympanic and trans-tympanic. Hence, we could not verify TympEALR by LA-TT-EALR to determine the false positive and false negative rates for TympEALR.

This study was performed only at our university hospital ORL department. We tested patients who met our inclusion criteria for CI and in addition for tympanic promontory testing. A future multicenter approach could enlarge the number of patients, add more experiences about difficulties when performing TympEALR, and reduce potential biases like patient selection bias.

## 6. Conclusions

The tympanic pre-operative electrically evoked auditory late response (= TympEALR) resulted in clear positive results in seven of the sixteen cases. Assuming that positive TympEALR does avoid invasive procedures like trans-tympanic promontory testing, we avoided such a procedure in almost half the patients of our study group. Though not reaching the reliability of trans-tympanic electrically evoked auditory potential measurements due to the distance between the stimulation electrode and cochlear nerve and resulting problems of co-stimulation and also in recording responses, TympEALR could avoid the need for its trans-tympanic alternatives in many with a positive response. If a clear negative TympEALR may speak against cochlear implantation should be investigated in further studies. Based on this study’s data, TympEALR can be used as a screening test whether a CI will work in a patient or not. Together with, e.g., radiology data like CT or MRI and the willingness of patients to be implanted, TympEALR can be a helpful tool in CI candidacy check. A differentiation between poor and good performers amongst positive TympEALR is currently not possible and needs to be investigated in a future study.

## Figures and Tables

**Table 1:** Clinical standard recording values for electrically evoked late response (EALR) recording.

Recording Parameter	EALR Value
Bandpass filtering	1 to 100 Hz
Monitoring time	1000 ms
Analysis time	750 ms
Sweeps per curve	30
Number of curves per level	2 to 3
Rejection level	±100 µV

Hz = Hertz; ms = milliseconds; µV = microvolts.

**Table 2:** Overview of the patients’ history of hearing.

Pat. Number	Age [y]	Tested Ear	Etiology of Hearing Loss Resp. Deafness	Duration of Hearing Loss [y]	Duration of Deafness [y]
1	34	left	Meningitis	23	14
2	72	left	Unknown	63	Unknown
3	47	Right	Unknown	47	47
4	16	Right	Unknown	16	16
5	62	Right	VS resection	18.5	18.5
6	47	Left	VS	1	1
7	43	Left	Unknown	10	10
8	32	Left	Unknown	0.25	0.25
9	77	Left	Herpes Zoster	1.5	1,5
10	50	Left	Unknown	1	1
11	59	Left	Traffic accident	0.75	0.75
12	62	Left	Unknown	1.75	1
13	50	Right	VS	1	1
14	27	Right	Unknown	27	27
15	17	Right	Meningitis	16.5	16.5
16	56	Left and right	Legionella, Coma, Antibiotics	1	1

VS = vestibular schwannoma.

**Table 3:** Overview of the patients’ subjective sensations.

Kind of Sensation	Frequency ****	Most Frequent Location	Other Locations
None	0	-	-
Hearing *	2	-	-
Unknown **	12	100% in the ear	-
Pain	2	100% in the ear	-
Uncomfortable	3	100% in the ear	-
Other sensation ***	6	100% in the ear	-

* Patient reported clear subjective hearing. ** Patient was unsure how to describe the sensation. *** The patient’s sensation did not fit into the other given categories. **** The patients partly reported multiple sensations.

**Table 4:** Overview of the patients’ TympEALR regarding response amplitude and latencies of waveform markers. As patient 16 was tested in both ears the values for the right and left ear TympEALR are both listed.

Pat. Number	N1-P2/P3 [µV]	N1 [ms]	P2 [ms]	P3 [ms]	N2 [ms]
1	0.71	59.72	113.89	No Resp.	195.83
2	5.20	98.78	125.61	185.37	240.24
3	2.08	93.50	149.50	No Resp.	251.00
4	1.89	83.33	131.25	162.50	233.33
5	2.56	88.24	131.37	156.86	188.24
6	5.20	95.00	168.00	No Resp.	269.00
7	0.41	131.50	164.50	185.00	206.50
8	3.70	91.00	108.00	No Resp.	166.00
9	8.90	120.00	172.00	No Resp.	188.89
10	2.80	96.00	200.50	No Resp.	349.50
11	3.20	112.00	229.50	No Resp.	351.50
12	No Resp.	No Resp.	No Resp.	No Resp.	No Resp.
13	3.00	58.50	109.00	180.50	254.00
14	3.30	55.00	113.50	No Resp.	185.00
15	2.90	68.00	135.50	236.00	181.50
16Right 16Left	1.56 1.22	60.78 121.43	123.53 86.84	No Resp. 171.43	172.55 257.14

No Resp. = No response detected in TympEALR waveforms.

## Data Availability

The raw data supporting the conclusions of this article will be made available by the authors upon request.
